# Thyroid and androgen receptor signaling are antagonized by μ‐Crystallin in prostate cancer

**DOI:** 10.1002/ijc.33332

**Published:** 2020-10-31

**Authors:** Osman Aksoy, Jan Pencik, Markus Hartenbach, Ali A. Moazzami, Michaela Schlederer, Theresa Balber, Adam Varady, Cecile Philippe, Pascal A. Baltzer, Bismoy Mazumder, Jonathan B. Whitchurch, Christopher J. Roberts, Andrea Haitel, Merima Herac, Martin Susani, Markus Mitterhauser, Rodrig Marculescu, Judith Stangl‐Kremser, Melanie R. Hassler, Gero Kramer, Shahrokh F. Shariat, Suzanne D. Turner, Boris Tichy, Jan Oppelt, Sarka Pospisilova, Sabrina Hartenbach, Simone Tangermann, Gerda Egger, Heidi A. Neubauer, Richard Moriggl, Zoran Culig, Georg Greiner, Gregor Hoermann, Marcus Hacker, David M. Heery, Olaf Merkel, Lukas Kenner

**Affiliations:** ^1^ Department of Pathology Medical University Vienna Vienna Austria; ^2^ Center for Biomarker Research in Medicine (CBmed) Graz Austria; ^3^ Department of Biomedical Imaging and Image Guided Therapy Medical University Vienna Vienna Austria; ^4^ Department of Molecular Sciences, Uppsala BioCenter Swedish University of Agricultural Sciences Uppsala Sweden; ^5^ Ludwig Boltzmann Institute Applied Diagnostics Vienna Austria; ^6^ Department for Pharmaceutical Technology and Biopharmaceutics University of Vienna Vienna Austria; ^7^ School of Pharmacy University of Nottingham Nottingham UK; ^8^ Department of Laboratory Medicine Medical University Vienna Vienna Austria; ^9^ Department of Urology Medical University Vienna Vienna Austria; ^10^ Division of Urology, Department of Special Surgery Jordan University Hospital, The University of Jordan Amman Jordan; ^11^ Institute for Urology and Reproductive Health Sechenov University Moscow Russia; ^12^ Departments of Urology Weill Cornell Medical College New York New York USA; ^13^ Department of Urology University of Texas Southwestern Dallas Texas USA; ^14^ Department of Urology, Second Faculty of Medicine Charles University Prague Czech Republic; ^15^ Division of Cellular and Molecular Pathology, Department of Pathology University of Cambridge Cambridge UK; ^16^ Center of Molecular Medicine, Central European Institute of Technology Masaryk University Brno Czech Republic; ^17^ Histo Consulting Inc. Ulm Germany; ^18^ Department of Pathology Rudolfinerhaus Privatklinik Gmbh Vienna Austria; ^19^ Unit for Laboratory Animal Pathology University of Veterinary Medicine Vienna Vienna Austria; ^20^ Institute of Animal Breeding and Genetics University of Veterinary Medicine Vienna Vienna Austria; ^21^ Department of Urology Innsbruck Medical University Innsbruck Austria; ^22^ MLL Munich Leukemia Laboratory Munich Germany; ^23^ Christian Doppler Laboratory for Applied Metabolomics (CDL‐AM) Medical University of Vienna Vienna Austria; ^24^ Present address: Jan Pencik, Molecular and Cell Biology Laboratory The Salk Institute for Biological Studies La Jolla California USA

**Keywords:** μ‐Crystallin, androgen receptor, prostate cancer, PSMA‐PET, thyroid hormone receptor

## Abstract

Androgen deprivation therapy (ADT) remains a key approach in the treatment of prostate cancer (PCa). However, PCa inevitably relapses and becomes ADT resistant. Besides androgens, there is evidence that thyroid hormone thyroxine (T4) and its active form 3,5,3′‐triiodo‐l‐thyronine (T3) are involved in the progression of PCa. Epidemiologic evidences show a higher incidence of PCa in men with elevated thyroid hormone levels. The thyroid hormone binding protein μ‐Crystallin (CRYM) mediates intracellular thyroid hormone action by sequestering T3 and blocks its binding to cognate receptors (TRα/TRβ) in target tissues. We show in our study that low CRYM expression levels in PCa patients are associated with early biochemical recurrence and poor prognosis. Moreover, we found a disease stage‐specific expression of CRYM in PCa. CRYM counteracted thyroid and androgen signaling and blocked intracellular choline uptake. CRYM inversely correlated with [18F]fluoromethylcholine (FMC) levels in positron emission tomography/magnetic resonance imaging of PCa patients. Our data suggest CRYM as a novel antagonist of T3‐ and androgen‐mediated signaling in PCa. The role of CRYM could therefore be an essential control mechanism for the prevention of aggressive PCa growth.

## Abstract 2

**What's new?**

Thyroid hormones may play a role in the progression of prostate cancer (PCa). In this study, the authors found that PCa cells had decreased expression of the thyroid‐hormone‐binding protein μ‐Crystallin (CRYM). Lower CRYM was also associated with poor prognosis in men with PCa. In addition, CRYM inhibited thyroid‐hormone and androgen signaling, as well as 18F‐fluoromethylcholine uptake by PCa cells. These results suggest that CRYM may act as a novel antagonist against factors that fuel PCa progression, and therefore thyroid signalling may offer a new therapeutic target in slowing aggressive prostate cancer.

**Abbreviations**
[^125^I]‐T3: triiodothyronine (T3) labeled with radioactive ^125^I
^1^H‐NMR: proton nuclear magnetic resonance
ADT: androgen deprivation therapy
AR: androgen receptor
ATCC: American Type Culture Collection
BCL3: B‐cell leukemia 3
BCR: biochemical recurrence
CHKA: choline kinase α
CMV: cytomegalovirus
CRPC: castration‐resistant prostate cancer
CRYM: μ‐Crystallin
CS‐FCS: charcoal‐stripped fetal calf serum
DHT: dihydrotestosterone
DIO1: iodothyronine deiodinase 1
DIO3: iodothyronine deiodinase 3
FASN: fatty acid synthase
FM: full medium
FMC: [18F]fluoromethylcholine
G‐418: geneticin
GFP: green fluorescent protein
GL3: Gleason pattern 3
GL4: Gleason pattern 4
HFM: hormone‐free medium
IHC: immunohistochemistry
IPA: ingenuity pathways analysis
MCT8: monocarboxylate transporter 8
PBS: phosphate‐buffered saline
PCA: principal component analysis
PCa: prostate cancer
PET: positron emission tomography
PET/MRI: positron emission tomography‐magnetic resonance imaging
PLS‐DA: partial least squares‐discriminant analysis
PSA: prostate‐specific antigen
PTEN: phosphatase and tensin homolog protein
PTU: propylthiouracil
qPCR: quantitative polymerase chain reaction
RB: retinoblastoma protein
RIPA: radioimmunoprecipitation assay
RXR: retinoid X receptor
T3: 3,5,3′‐triiodo‐l‐thyronine
T4: thyroid hormone thyroxine
TH: thyroid hormone
TMAs: tissue microarrays
TRα: thyroid hormone receptor alpha
TRβ: thyroid hormone receptor beta
TTR: transthyretin
VIP: variable importance for projection

## 1 INTRODUCTION

Prostate cancer (PCa) is the most frequently diagnosed cancer in men in the Western world. The course of PCa is largely driven by androgen receptor (AR) and, consequently, androgen ablation therapy is a cornerstone of current therapies in advanced stages of the disease.^1^, ^2^ A large proportion of patients with advanced PCa become resistant to androgen deprivation therapy (ADT) resulting in lethal castration resistant prostate cancer (CRPC). CRPC is mainly associated with genetic amplifications, mutations or other aberrations in the AR.^3^, ^4^ Besides androgens, the role of thyroid hormone thyroxine (T4) and its more active form 3,5,3′‐triiodo‐l‐thyronine (T3) in the progression of PCa has not been comprehensively elucidated.

The thyroid hormones released into circulation are mostly bound to plasma proteins and subsequently transported to the cytosol by thyroid hormone (TH) transporters, which have diverse binding affinities such as transthyretin (TTR) and thyroxine‐binding globulin.^5^ The activity of TH within the cell is regulated by (a) cell uptake involving transporters such as MCT8, (b) metabolization by DIO1 and DIO3, two members of the iodothyronine deiodinase family, and (c) sequestration via binding to other proteins^6^ such as the cytoplasmic protein μ‐Crystallin (CRYM) which is known to bind to T3 with high‐affinity.^7^ CRYM is related to an enzyme involved in amino acid metabolism, ornithine cyclodeaminase,^8^ which plays an important role in PCa tumorigenesis via AR signaling.^9^ Intracellular thyroid hormone function in the prostate is dependent on CRYM expression levels.^10^, ^11^ Previous reports demonstrated that CRYM expression was downregulated in PCa patients who underwent ADT^12^ and that expression of CRYM was also downregulated in a PCa xenograft tumor model.^13^ It was shown that CRYM expression was particularly low in therapy refractory PCa patient biopsies as compared to with primary tumors.^13^ We corroborated this finding and showed that CRYM was responsive to androgens in the MDA PCa 2b cell line.^12^

The objective of our study was to define the role of thyroid hormone and its regulation by CRYM in PCa. We hypothesized that TH signaling might play an auxiliary role in PCa progression. Here we show that CRYM expression levels are lower in PCa compared to normal prostate tissue and are reduced further in metastatic disease. Moreover, Kaplan‐Meier analysis reveals low CRYM expression as a negative prognostic factor. In PCa cell lines, CRYM expression significantly suppresses thyroid hormone‐ and androgen‐induced genes. Intracellular [18F]‐fluoromethylcholine uptake in PCa was also significantly blocked by CRYM. Importantly, [18F]fluoromethylcholine (FMC) positron emission tomography (PET)/magnetic resonance imaging (MRI) in vivo imaging of PCa patients correlates inversely with CRYM levels. Our data propose a novel role for CRYM as a strong antagonist of thyroid and androgen hormone pathways. The important function of CRYM could be a control mechanism for the progression of PCa.

## 2 MATERIALS AND METHODS

### 2.1 Cell lines

PCa cell lines LNCaP (RRID: CVCL_0395), PC3 (RRID: CVCL_0035), DU145 (RRID: CVCL_0105) and 22Rv1 (RRID: CVCL_1045) were obtained from American Type Culture Collection (ATCC) (Manassas, VA) and cultured in RPMI 1640 medium (Gibco Life Technologies, Carlsbad, CA) supplemented with 10% FCS (Gibco) and 1% penicillin/streptomycin (Gibco) at 37°C in 5% CO_2_ in a humidified chamber. VCaP (RRID: CVCL_2235) cells were purchased from the ATCC and maintained in DMEM (Gibco Life Technologies) supplemented with 10% FCS and 1% penicillin/streptomycin. For hormone sensitive experiments, charcoal‐stripped fetal calf serum (FCS) was used (CS‐FCS, Gibco Life Technologies). The human PCa cell line LAPC4 (RRID: CVCL_4744) was kindly provided by Zoran Culig (Innsbruck Medical University) and cultured in Iscove's Modified Dulbecco's Medium (Sigma‐Aldrich, St. Louis, MO) supplemented with 10% FCS, 1% penicillin/streptomycin. RWPE‐1 (RRID: CVCL_3791) was obtained from ATCC and grown in Keratinocyte serum‐free media with l‐glutamine (Invitrogen) supplemented with 2.5 mg EGF (Invitrogen) and 25 mg Bovine Pituitary Extract (Invitrogen). All cell lines were assessed to be free of mycoplasma infection and were passaged using trypsin/EDTA (Gibco). All human cell authentication was done by genetic profiling using polymorphic short tandem repeat loci (STRs) within the last 3 years.

### 2.2 Prostate cancer immunohistochemistry and human tissue microarray

Formalin‐fixed paraffin‐embedded samples were obtained from patients who underwent radical prostatectomy (Department of Pathology, Medical University Vienna, Austria, and Institute of Pathology, Tuebingen, Germany). Immunohistochemistry (IHC) was carried out according to standard protocols and protein expression quantified as described.^14^ The following antibodies were used in our study: CRYM (Cat# H00001428‐M03, clone 6B3, 1:100, Abnova), TRβ (Cat# 209‐301‐A96, 1:100, Rockland), AR (Cat# M3562, 1:250, DAKO), Ki‐67 (Cat# NCL‐Ki67p, Leica Microsystems), prostate specific antigen (PSA) (Cat# 2475S, 1:20, Dako), Cleaved Caspase 3 (CC3) (Cat# 9661, 1:200 dilution; Cell Signaling). Tissue microarrays (TMAs) were evaluated by four board certified pathologists (M. S., M. H., A. H. and L. K.) and statistical analysis was performed as described.^15^ Biochemical recurrence (BCR) was defined as PSA progression with ≥25% increase in PSA and PSA levels above 0.2 ng/mL. For BCR‐free survival analysis, Kaplan‐Meier survival curves and Log‐Rank tests were conducted. The following R/Biocondor packages were used: survival, survminer. TMAs were performed on the respective tumor areas as well as benign prostatic hyperplasia areas. After IHC staining for CRYM and TRβ as described above, TMAs were assessed by three pathologists (S. H., S. T. and L. K.) according to a 4‐point scale for staining intensity (0‐3) and a 4‐point scale for the number of stained cells (0‐3). A sum score (0‐6) served as the reference for correlation with semiquantitative FMC‐PET data. Patients received a routine clinical follow‐up monitoring using PSA serum blood sampling. Biochemical relapse was defined as PSA levels above 0.2 ng/mL. For initial staging, synchronous metastatic disease was defined by positive lymph node findings after surgery or biopsy of imaging positive distant lesions.

### 2.3 Oncomine database analysis

Gene expression analysis of *CRYM*, *AR* and *THRB* (TRβ) was performed in various prostate cancer datasets representing normal, tumor or metastatic samples deposited in the Oncomine Research Premium Edition database (Thermo Fisher, Ann Arbor, MI). For the analysis, the *P* value threshold was set to .05, the fold‐change threshold was set to 1.5 and the gene rank threshold was set to “all.”

### 2.4 Lentiviral transduction

To establish CRYM knock down LNCaP cell lines, cells were transduced with the Lentiviral particles targeting the human CRYM coding sequence (Mission shRNAlentiviral particles, Sigma) and a nontargeting shRNA construct as a control using a spinoculation procedure. Briefly, 1.5 × 10^4^ of LNCaP cells were seeded in a 96‐well plate and incubated in a humidified chamber at 37°C and 5% CO_2_ to accommodate a confluence of 70% to 80% on the day of transduction. The medium was removed and replaced with 110 μL fresh medium containing 8 μg/mL Polybrene (Sigma) added to each well to increase the transduction efficiency. A quantity of 10 μL of lentiviral particles was added to the appropriate wells and the cells and lentiviral particles containing plates were immediately centrifuged at 32°C for 90 minutes at 2500 rpm speed in a centrifuge (Beckman Allegra X‐12R). After spinoculation, plates were incubated for 24 to 48 hours at 37°C in a humidified chamber and cells were washed with phosphate‐buffered saline (PBS) to remove residual lentiviral particles. Stable clones of LNCaP cells were selected by adding 1 μg/mL of puromycin antibiotic (Millipore).

### 2.5 Colony formation

To examine the effect of T3 on colony formation, a bottom layer containing RPMI 1640 medium (10% CS‐FCS) and 0.75% low melting agar (Bio‐Rad) was overlain with half the volume of RPMI 1640 medium (10% FCS) and 0.36% agar containing 3 × 10^4^ cells. Finally, the two layers were covered with liquid RPMI 1640 medium containing 10% CS‐FCS (Gibco Life Technologies). The liquid top layer with or without 100 nM T3 was changed every 3 days. Cells were incubated at 37°C for 14 days. The top layer was discarded and the two remaining layers were flushed with PBS. Colonies were fixed and stained with crystal violet. The colonies were counted and photographed (Nikon D90).

### 2.6 Quantification of PSA and T3

Lentivirally transduced LNCaP cells were seeded at a density of 10^5^ cells/mL in 6‐well plates and were cultured in RPMI 1640 with 10% FCS for 48 hours. The culture medium was removed and replaced with RPMI 1640 medium with 10% CS‐FCS (Gibco Life Technologies) and cells were incubated with or without 50 nM T3 for another 3 days. The growth medium was collected at 24, 48 and 72 hours. For T3 analysis, PCa cells were transfected by lipofection with empty vector or CRYM expressing vector. After 48 hours, medium was replaced with RPMI medium with or without T3. The culture medium was collected at 24 and 48 hours. The level of secreted PSA and T3 in the growth medium was determined using a validated Elecsys electrochemiluminescence immune assay (Roche, Rotkreuz, Switzerland) on a Cobas e601system (Roche) that is routinely used for clinical applications.

### 2.7 Western blotting

Cells were lyzed using radioimmunoprecipitation assay (RIPA) buffer supplemented with a cocktail of protease inhibitors (Roche). Western blot analysis was performed using standard procedures. Protein lyzates were separated by SDS‐polyacrylamide gel electrophoresis and then transferred to polyvinylidene difluoride membrane. The membranes were blocked with 5% wt/vol nonfat dry milk and incubated with CRYM (Cat# H00001428‐M03, M03 clone 6B3, Abnova) and TRβ (Cat# 209‐301‐A96, clone 2386 IgG1, Rockland), AR (Cat# sc‐816, clone H1612, Santa Cruz Biotechnology), PSA (Cell Signaling Technology), Choline Kinase α (Cat# 13422, D5X95, Cell Signaling) antibodies at a dilution of 1:1000. β‐Actin (Cat# 4967, 1:10 000 dilution, Cell Signaling Technology) β‐tubulin (Cat# 5666, 1:10 000 dilution, Cell Signaling Technology). Secondary mouse (315‐0035‐008) and rabbit (111‐036‐047) antibodies were obtained from Jackson Immune Research. Densitometric analysis of Western blots was performed using ImageJ software and band intensities were normalized to the corresponding housekeeping gene β‐actin. All densitometric analyses for Western blots are shown in Figure S4A to H.

### 2.8 RNA isolation, cDNA and quantitative polymerase chain reaction

Total RNA was isolated from cultured cells homogenized in QIAzol Lysis Reagent (Qiagen, Germany) using a single‐step guanidine isothiocyanate/phenol/chloroform‐based extraction technique. RNA concentration and quality were measured using a NanoDrop 2000 UV‐Vis Spectrometer (Thermo Fisher Scientific, Wilmington, DE). Using RevertAid First Strand cDNA Synthesis Kit (ThermoFisher Scientific), 1000 ng of RNA were reverse‐transcribed to cDNA. For quantitative polymerase chain reaction (qPCR) analysis, CFX96 Real‐Time PCR Detection System (BioRad, Hercules, CA) was employed using Kapa Sybr Fast qPCR Master Mix (2x) Kit (Kapa Biosystems, Wilmington, MA). RNA expression of target genes relative to glyceraldehyde‐3‐phosphate dehydrogenase was quantified by ΔΔCT method. Statistical analysis was performed in GraphPad Prism 6.

### 2.9 Invasion assay

For invasion assays, matrigel‐coated invasion chambers (BD Biosciences, NJ) were rehydrated for 2 hours in RPMI 1640 medium (Gibco Life Technologies) supplemented with 10% FCS (Gibco). Cells (2.5 × 10^4^/mL) were seeded in the upper chamber in RPMI 1640/10% FCS, whereas the lower chamber contained RPMI 1680/15% FCS. After 24 hours at 37°C, medium was discarded and noninvaded cells were removed with cotton swabs from the top of the membrane, cells that migrated to the bottom of the membrane were fixed with methanol and stained with Toluidine blue. Invading cells were counted using a microscope equipped with a camera.

### 2.10 Gene expression profiling

Total RNA was extracted using TRIzol Reagent (Ambion, Thermo Scientific). RNA‐Seq libraries were prepared with a TruSeq Stranded mRNA LT sample preparation kit (Illumina) using Sciclone and Zephyr liquid handling robotics (PerkinElmer). For sequencing, libraries were pooled, diluted and sequenced on an Illumina Hi‐Seq 2000 using 50 bp single‐read v3 chemistry. Transcriptome analysis was performed using the Tuxedo suite. TopHat2 was supplied with reads passing vendor quality filtering (PF reads) and the Ensembl transcript set (*Homo sapiens*, e73, September 2013) as reference. Differential expression was assessed with Cuffdiff v2.1.1. The experiment was performed in triplicates. Fragments per kilo bases of exons per million mapped reads values and fold changes of significantly upregulated and downregulated genes are shown in Figure S2A. Significantly deregulated genes (*P* < .05, >2‐fold, n = 642) were analyzed using ingenuity pathway analysis (IPA) to identify upstream regulators of deregulated genes (enrichment of target genes as reflected in *P* value) and activation or inhibition of the respective pathway, reflected in a positive or negative *z* score, respectively. A tight interaction of thyroid and androgen signaling was observed (circles represent target genes of the respective pathway: Progesterone receptor, vascular endothelial growth factor, androgen receptor [AR], dihydrotestosterone [DHT]). Of 58 540 assessed RNA types, 34 878 (59.6%) were expressed in LNCaP cells. CRYM overexpression led to significant deregulation in 9.25% of these (2.72% up, 6.53% down; >2‐fold).

### 2.11 Cell proliferation

LNCaP and PC3 cells were seeded into 6‐well plates in triplicates at a density of 10^5^ cells/mL in RPMI 1640 medium (supplemented with 10% FCS). After 24 hours, cells were washed with PBS and medium was replaced with Charcoal‐stripped RPMI 1640 medium (Gibco). 3,3′,5‐Triiodo‐l‐thyronine (T3) were purchased from Sigma‐Aldrich and dissolved in Dimethylsulfoxide (DMSO) at a concentration of 100 mM (T3). Growth medium of LNCaP cells was supplemented with 0.1 to 100 ng/mL of T3 and the cells were grown for 3 days. PC3 cells were supplemented with 100 ng/mL of T3 and were grown for 3 days. After trypsinization and collection by centrifugation, cells were resuspended and counted using a hemocytometer (C‐chip).

### 2.12 Transfection of cells

CRYM expressing cells were generated by transfecting PCa cells with pReceiver‐M72 Empty Vector and pReceiver‐M72 CRYM (for simplicity referred to in the text as CRYM(+) (GeneCopoeia) containing a green fluorescent protein (GFP) reporter gene using Lipofectamine LTX reagent (Invitrogen, Carlsbad, CA) according to the manufacturer's protocols. GFP expression was evaluated under a fluorescent microscope and cells were selected using 500 μg/mL of G‐418 antibiotic for 4 days (Gibco). Cells used for analysis were >70% GFP‐positive. Transfection efficiency was confirmed by direct observation of GFP expression at 3 days post‐transfection and RNA‐seq with total RNA was performed at this time point.

### 2.13 [^125^I]T3 uptake experiments

Cell culture medium (RPMI, 10% FCS) used in LNCaP and PC3 cells overexpressing CRYM or empty vector was discarded and replaced with 1 mL of charcoal‐stripped RPMI per well. Cells were incubated with 45 kBq l‐3,5,3′‐[^125^I]‐triiodothyronine (PerkinElmer) for 12, 24 and 48 hours in an incubator (humidified atmosphere, 37°C, 5% CO_2_). After incubation, the supernatant was taken off the cells. Cells were washed with PBS, trypsinized and centrifuged. Radioactivity was measured in all fractions using a gamma‐Counter (2480 WIZARD^2^, PerkinElmer) and uptake values were calculated as percentage of applied dose per 1 × 10^5^ cells (%AD/1 × 10^5^ cells).

### 2.14 NMR‐based metabolomics analysis of culture media and cell pellets

The culture media from PC3 and RWPE‐1 were filtrated using 3‐kDa cutoff Nanosep centrifugal filters (Pall Life Science) to remove cell debris and macromolecules prior to NMR analysis.^16^, ^17^ In order to remove glycerol from the filter membrane, filters were washed eight times with 500 μL H_2_O at 36°C shaking at 400*g* speed and followed by filtering of 500 μL of culture medium at 4°C at 10 000*g*. Phosphate buffer (150 μL, 0.4 mol/L, pH 7.0), D_2_O (45 μL) and TSP (30 μL, 5.8 mmoL/L) (Cambridge Isotope Laboratories) as internal standard were added to 375 μL of culture media filtrate. The mixture was then used for NMR analysis (5 mm NMR tube). The cell pellet collected from culture media of PC3 and RWPE‐1 were extracted with 1 mL of methanol taken directly from the freezer (−20°C). After centrifugation at 10 000*g* speed and 4°C, the supernatant was taken and dried using nitrogen. The samples were then dissolved in a mixture containing phosphate buffer (520 μL, 0.135 mol/L), D_2_O (50 μL) and TSP (30 μL, 5.8 mmoL/L) (Cambridge Isotope Laboratories) as internal standard and were analyzed by NMR. The complete sets of NMR quantifications were carried out using a Bruker spectrometer at 600 MHz. ^1^H NMR spectra were obtained using the zgesgp pulse sequence at 25°C, with 200 scans for culture media and 400 scans for cell pellet extract, 65 536 data points over a spectral width of 17 942.58 Hz. Acquisition duration was selected as 1.82 seconds, and relaxation delay was chosen as 4.0 seconds. Bruker Topspin 1.3 software was used to run the NMR spectra and NMR was transformed following multiplication by enlarging of 0.3 Hz. NMR spectra were adjusted to TSP at 0 ppm and baseline together with spectral phase was adjusted. Thirty‐nine metabolites on the NMR spectra of the culture media were identified and quantified by manually integrating specific spectral regions after accounting for overlapping signals using NMR Suite 7.1 profiler (ChenomX, Inc., Edmonton, Canada). A list of metabolites quantified, their corresponding NMR signals used for integration and quantification, and the order of integration, in order to account for overlapping signals, are presented in Figure S3A. NMR analysis for cell extracts was performed by integrating NMR spectra into 0.01‐ppm integral regions (buckets) and residual H_2_O was removed. The identification of NMR signals was carried out using NMR 7.1 library (ChenomX, Inc.), Biological Magnetic Resonance Data Bank and Human Metabolome Data Base.

### 2.15 Multivariate data analysis

The SIMCA‐P+ 13.0 software (Umetrics, Umeå, Sweden) was used to perform multivariate data analysis, principal component analysis (PCA) and partial least‐squares‐discriminant analysis (PLS‐DA) as described in.^16^ PCA and PLS‐DA models were fitted using metabolomics data from culture media or cell pellet extract of PC3 cells and RWPE‐1 with and without CRYM vector and T3 treatments. For the PLS‐DA models were fitted using metabolomics data from culture media and cell pellet extracts, the variable importance for projection (VIP) for each metabolite or spectral regions were implemented to determine the discriminative metabolites or spectral regions onward the first two components. A metabolites region with VIP ≥ 1 and the corresponding jackknife‐based 95% confidence interval (CI) ≤ 0.5 (VIP − CI ≥ 0.5) were considered discriminative. The VIP (CI) of all 39 metabolites (measured in the culture media) along the first and second components of the PLS‐DA model are presented in the Figures 5A and S3A. For the cell pellet extract, first the spectral regions with VIP − CI ≥ 0.5 were determined and identified (as described above). For those metabolites whose NMR signals appear in a different spectral region, the VIP (CI) of the spectral region with the highest VIP − CI and minimum spectral overlap with other metabolites was chosen for further presentation as relative concentrations.

### 2.16 FMC‐PET/MRI patient study

A subgroup of 87 patients out of the preoperative study training cohort was eligible for the presented subanalyses. All patients underwent FMC‐PET/MRI for primary staging. Patients gave their written informed consent. Finally, 42 patients underwent radical prostatectomy and postsurgery whole mount sections were available for analyses. The ongoing clinical trial is conducted according to International Council for Harmonisation‐Good clinical practice and the Declaration of Helsinki.

### 2.17 Statistical analyses

After testing for equal distribution of values, a Student's *t* test was used for comparison of groups. If no equal distribution was found, we used a Mann‐Whitney *U* test. For calculation of Kaplan‐Meier curves, we used IBM SPSS Statistics Version 22 with the survival package of R (R Development Core Team, 2009), an open‐source language and environment for statistical computing, and *P* values were calculated using a log‐rank test. Statistical methods of survival analyses, as well as follow‐up details of our study population have been described in publication.^18^ We conducted statistical analyses using the R environment v3.5.1 (https://cran.r-project.org). All immunoblot experiments were repeated two to three times, and the data were expressed as the mean ± SD. Statistical analysis was performed by the Student's *t* tests for comparing two groups and by analysis of variance for multiple group comparisons; *P* < .05 was considered statistically significant.

## 3 RESULTS

### 3.1 Low μ‐Crystallin (CRYM) expression is a negative prognostic factor and a hallmark of PCa

**FIGURE 1:**
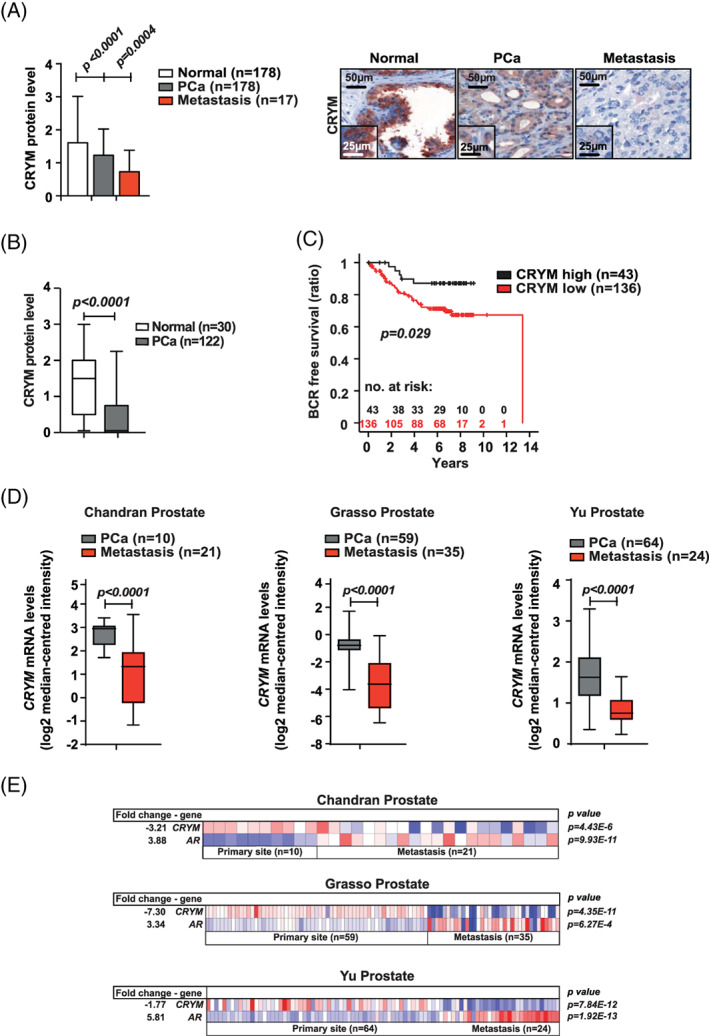
CRYM is stage‐dependently expressed in prostate cancer at the mRNA and protein level. A, CRYM protein levels assessed by IHC of healthy prostate tissue (n = 178), PCa (n = 178) and tissues derived from PCa metastases (n = 17). Representative staining for CRYM in healthy prostate, PCa and metastatic tissue is shown, scale bar 50 μm (low magnification), 25 μm (inlet, high magnification). B, IHC analysis of CRYM protein levels in non‐neoplastic prostate tissue (n = 30) and PCa (n = 122) in an independent cohort from Tuebingen. C, IHC CRYM protein levels in correlation to time for BCR in a Kaplan‐Meier analysis (*P* = .029). D, *CRYM* mRNA expression levels in PCa patients, analyzed from primary tumor samples and metastases. Data were extracted from the Oncomine Platform from the following studies: Chandran Prostate (left, *P* < .0001), Grasso Prostate (middle, *P* < .0001) and Yu Prostate (right, *P* < .0001). E, Heatmaps of human patient data depicting *CRYM* and *AR* mRNA levels in primary tumors and PCa metastases (log2 median‐centered intensity). Data were extracted from the Oncomine Platform from the following studies: Chandran Prostate (upper), Grasso Prostate (middle) and Yu Prostate (lower). BCR, biochemical recurrence; CRYM, μ‐Crystallin; IHC, immunohistochemistry; PCa, prostate cancer [Color figure can be viewed at wileyonlinelibrary.com]

We assessed CRYM expression levels in malignant and adjacent normal biopsies derived from a large PCa patient cohort by IHC. Decreased protein expression of CRYM was observed in PCa patient samples (n = 178) compared to normal prostate glands (n = 178), and a further reduction in CRYM expression was observed in metastatic samples (n = 17, Figure 1A). Representative IHC images of are shown (Figure 1A). These findings were validated through a second independent cohort covering PCa and benign prostate tissue samples (Tuebingen cohort, Figure 1B). PCa specimens (n = 122) had lower CRYM protein levels as compared to benign samples (n = 30). We analyzed the effect of CRYM expression on BCR‐free survival by Kaplan‐Meier analysis in 179 PCa Vienna cohort patients (Figure 1C). Low CRYM expression was associated with a reduced time to BCR, indicating that low CRYM expression is associated with poor prognosis. To validate whether *CRYM* gene expression was associated with PCa progression, we analyzed a large collection of independent datasets from the Oncomine database.^19^ As shown in Figure 1D, three datasets, Chandran, Grasso and Yu prostate cancer cohorts, clearly showed that *CRYM* expression was significantly downregulated in metastases compared to the levels in primary PCa (*P* < .0001). Analysis of two additional data sets, Varambally and LaTulippe, also showed that *CRYM* was higher expressed in normal tissues or primary PCa compared to metastases (Figure S1A). Furthermore, Figure S1B shows that prostate tumors with low Gleason score and stage (VI, N0) expressed a higher level of *CRYM* mRNA as compared to tumors with higher Gleason score and pathological stage (VII and IX, N1+) (Vanaja cohort; *P* = .00024, Luo 2 cohort; *P* = .007 and LaTullipe cohort; *P* = .015). Remarkably, we identified that *AR* and *CRYM* expression levels showed an inverse correlation in Chandran, Grasso and Yu cohorts (Figure 1E). Expression heatmaps show significant upregulation of *AR* in patients where *CRYM* expression is reduced in metastases compared to primary PCa samples (Figure 1E). Overall, these data indicate that loss of CRYM expression in PCa is an indicator for PCa aggressiveness. Taken together, the expression and prognostic value of CRYM suggests a role in regulation of AR and TH signaling in PCa.

### 3.2 CRYM enables intracellular accumulation of T3 in PCa cells

**FIGURE 2:**
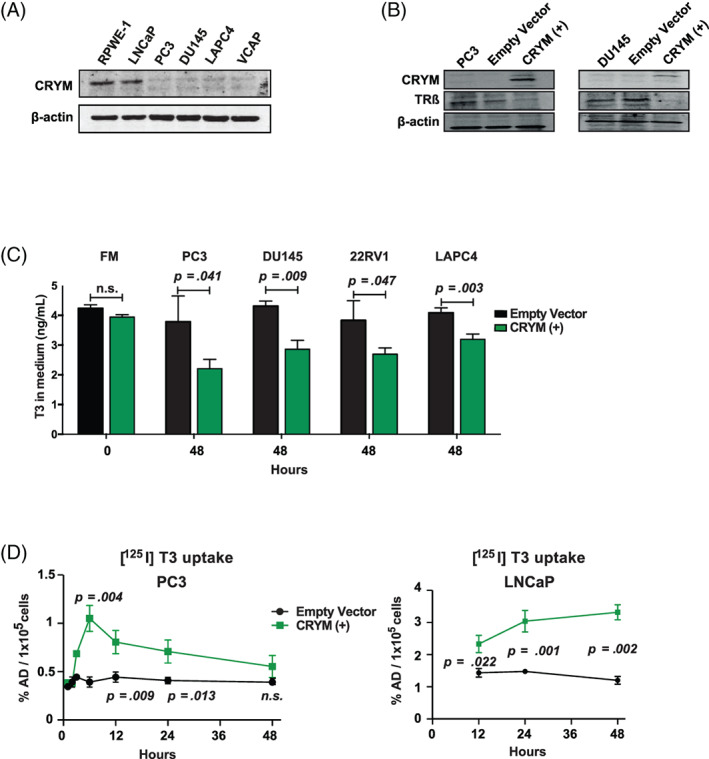
CRYM overexpression leads to a reduction of free T3. A, Immunoblot analysis of CRYM and TRβ in PCa cell lines RWPE‐1, LNCaP, PC3, DU145, LAPC4, VCAP. β‐Actin was used as loading control. B, CRYM and TRβ levels in PC3 and DU145 cells that were transiently transfected with EV or CRYM(+). C, Human PCa cells PC3, DU145, 22Rv1 and LAPC4 were transfected with a plasmid bearing the CRYM insert under the CMV promoter that coexpresses GFP (CRYM(+)) or empty vector (EV). T3 concentrations were determined across human PCa cells by analyzing with electrochemiluminescence immune assay (n = 3). FM (Full medium): RPMI medium supplemented with 10% FCS. D, Transiently transfected PC3 and LNCaP cells with EV or CRYM(+) were incubated with radioactively labeled T3 ([^125^I] T3) in hormone free medium for 48 hours, and intracellular radioactivity was determined by scintillation counting. CRYM, μ‐Crystallin; FCS, fetal calf serum; PCa, prostate cancer [Color figure can be viewed at wileyonlinelibrary.com]

**FIGURE 3:**
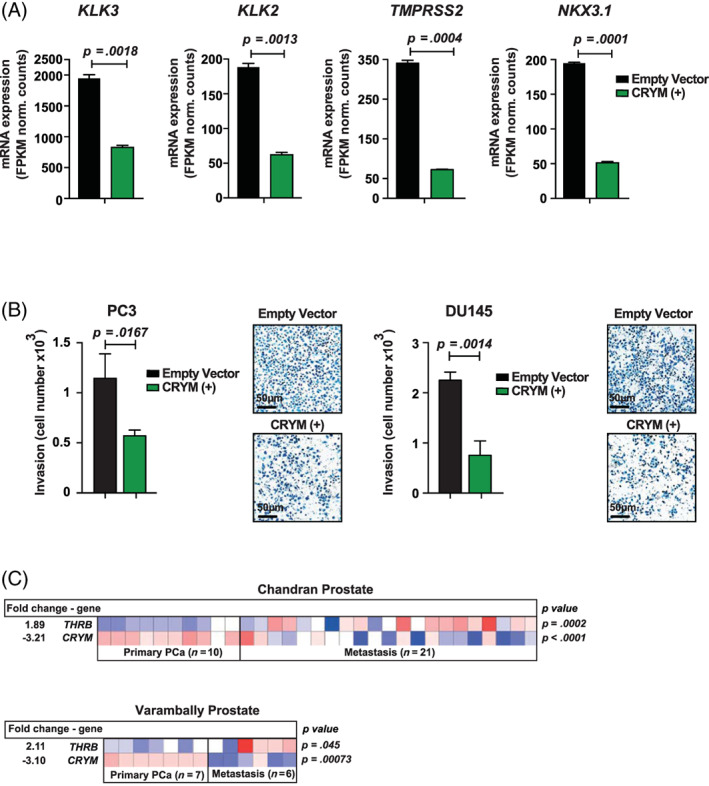
CRYM overexpression represses AR signaling and invasion in PCa cells. A, RNA‐seq analysis of LNCaP cells that were transiently transfected with EV or CRYM(+). AR responsive genes *KLK3* (PSA) (*P* = .0018), *KLK2* (*P* = .0013), *TMPRSS2* (*P* = .0004) and *NKX3.1* (*P* = .0001) are shown. B, Matrigel‐coated invasion chambers were used to test the invasive capacity of PCa cell lines PC3 and DU145 with and without CRYM(+) over 24 hours. Quantification and representative images are shown; scale bar = 50 μm. C, Heatmaps depicting *THRB* (TRβ) and *CRYM* mRNA levels in primary PCa and metastatic patient samples (log2 median‐centered intensity). Data were extracted from the Oncomine Platform from the Chandran Prostate (upper) and Varambally Prostate (lower) studies. AR, androgen receptor; CRYM, μ‐Crystallin; EV, empty vector; PCa, prostate cancer [Color figure can be viewed at wileyonlinelibrary.com]

WB analyses have shown endogenous CRYM expression in androgen‐dependent PCa cell lines RWPE‐1 and LNCaP (Figure 2A), while endogenous TRβ expression was present in all PCa cell lines and only showed reduced expression in the nontransformed prostate cell line RWPE‐1. Furthermore, WB analysis confirmed the androgen‐dependent expression of PSA (KLK3) in PCa cell lines (Figure S1C). Reintroduction of CRYM expression in PC3 and DU145 cells reduced TRβ expression (Figure 2B). To determine the effect of CRYM on intracellular accumulation of T3, we transfected CRYM negative PCa cell lines with empty vector or CRYM(+) expression vector (Figure 2C). Growth medium was supplemented with 10 nM T3 or vehicle, and after 48 hours, T3 levels were measured in the supernatants using an immune‐chemiluminescence assay. We observed significantly reduced levels of T3 in the medium of all CRYM re‐expressing cells (PC3, 44%; DU145, 22%; 22RV1, 20% and LAPC4, 18%) compared to controls Figure 2C. However, T3 uptake measured in the growth medium of LNCaP did not significantly change at 48 hours (Figure S1D). To confirm that this reflected uptake into cells, radioactively labeled thyroid hormone [^125^I] T3 was supplemented to the culture media. More T3 was found in PC3 and LNCaP cell lines with CRYM overexpression compared to empty vector controls (Figure 2D). It can be hypothesized that CRYM binds free T3 in the tumor cells, which results in increased T3 uptake and concomitantly reduced T3 levels in the growth medium. To assess the transcriptional response of CRYM overexpression in LNCaP cells, we performed RNA‐Seq analysis using empty vector or CRYM(+). Consistent with previous results,^12^ CRYM overexpression broadly inhibited genes involved in androgen signaling. Specifically, *KLK3*, *KLK2*, *TMPRSS2* and *NKX3.1*, which are affected by DHT, were suppressed by CRYM overexpression (Figure 3A). These results indicate that CRYM plays a major role in the AR gene expression program necessary for progression of metastatic PCa.

### 3.3 CRYM interferes with T3 signaling and reduces the invasive potential of PCa cells

We monitored the effect of high CRYM expression on invasive potential using matrigel‐coated invasion chambers. CRYM overexpression significantly reduced invasion by 50% (*P* = .0167) and 67% (*P* = .0014) for PC3 and DU145 cells, respectively (Figure 3B). Growth inhibition by CRYM overexpression was also examined in PC3 cells. Cells were transfected with empty vector or CRYM(+) and cell numbers were quantified at Day 2 and Day 4. Figure S1E shows that CRYM(+) led to inhibition of cellular proliferation after 4 days (*P* = .0004). Notably, in metastases *CRYM* mRNA expression was significantly downregulated (Chandran cohort: 3.21‐fold, *P* < .0001; Varambally cohort: 3.10‐fold, *P* = .0007) and *THRB* (TRβ) mRNA levels were concomitantly overexpressed (Chandran cohort: 1.89‐fold, *P* = .0002; Varambally cohort: 2.11‐fold, *P* = .045) compared to primary PCa tumor tissue (Figure 3C). Taken together, our data suggest that CRYM binds and sequesters T3 in PCa and correlates with reduced invasive capacity. Since free T3 is known to influence TRβ expression,^20^ we hypothesize an autoregulatory loop where CRYM masks free T3 and therefore leads to reduced TRβ expression.

### 3.4 CRYM re‐expression changes the transcriptome revealing thyroid and androgen receptor antagonism

**FIGURE 4:**
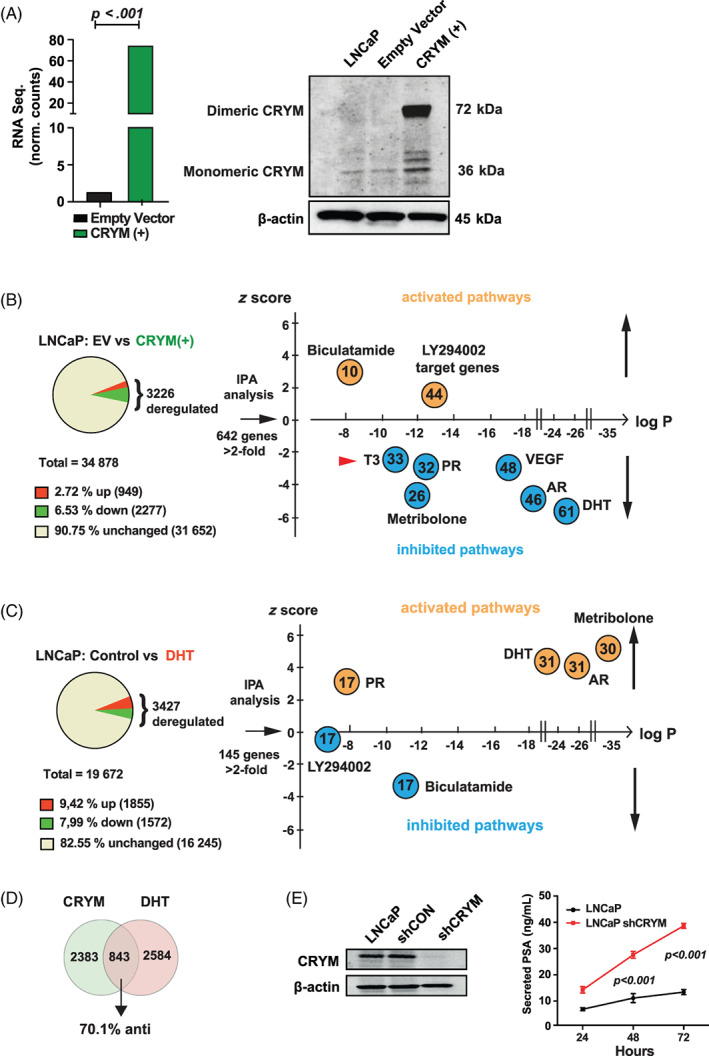
CRYM‐induced suppression of T3 and AR signaling. A, LNCaP cells expressing empty vector (EV) or CRYM(+) were analyzed by RNA‐seq and Immunoblot. B, LNCaP CRYM(+) and EV cells were compared using three biological replicates for each group. Single end 50 bp RNA‐seq was performed with an Illumina Hi‐Seq2000 platform and LNCaP CRYM(+) to EV cells were compared using three biological replicates for each group. CRYM overexpression was validated by RNA‐seq (73.7 vs 2.2 normalized counts, *P* < .001). Shown are enriched pathways according to IPA pathway analysis among the deregulated genes. C, Genes deregulated by androgen DHT in the same cell line LNCaP from a published dataset. Shown are enriched pathways according to IPA pathway analysis.^24^ D, 843 genes overlapped among CRYM overexpression and DHT regulated genes; 70.1% of these were counter‐regulated (anti). Androgen‐responsive genes and AR‐regulated genes are enriched in LNCaP EV vs CRYM(+) and control vs DHT. E, CRYM knockdown in the LNCaP cell line generated using lentiviral‐transfected shRNAs (shCRYM) and a nontargeting control shRNA (shControl). CRYM reduction was confirmed by Immunoblot. PSA release levels were measured by a chemiluminescence immune assay. AR, androgen receptor; CRYM, μ‐Crystallin; DHT, dihydrotestosterone; EV, empty vector; IPA, ingenuity pathways analysis; PSA, prostate‐specific antigen [Color figure can be viewed at wileyonlinelibrary.com]

Given the proposed antagonistic role of CRYM on thyroid hormone signaling and its negative association with advanced PCa,^11^, ^13^ we assessed the impact of CRYM overexpression on gene expression in androgen sensitive and AR‐expressing LNCaP cells using polyA enriched RNA‐sequencing (RNA‐Seq). Cells were transfected with a plasmid conferring neomycin resistance containing the CRYM ORF in addition to a GFP reporter under the cytomegalovirus (CMV) promoter, or empty vector control, and were selected in G‐418 containing medium (Figure 4A). Exogenous CRYM resulted in differential expression of 9.25% of genes (n = 3226; 2.72% up, 6.53% down, Figure 4B). IPA was performed on significantly deregulated genes (>2‐fold; *q* < 0.05; n = 642) to identify over‐represented pathways (see Table S1 for the detailed list of genes). Selected genes known to be activated by the TR/RXR (n = 33) axis were suppressed upon CRYM overexpression, including B‐cell leukemia 3 (*BCL3*) and fatty acid synthase (*FASN*). High *FASN* expression is a known feature of aggressive PCa and it has been proposed as a metabolic oncogene.^21^, ^22^ Interestingly, also genes involved in AR signaling including DHT (n = 61) and AR regulated genes (AR; n = 46) were significantly downregulated (Figure 4B).

We then compared our RNA‐Seq data set of CRYM overexpressing cells to a published RNA‐Seq data set^23^ wherein the same cell line had been treated with DHT (Figure 4C). We performed IPA analysis on the DHT deregulated genes (>2‐fold, *q* < 0.05; n = 145). As expected, progesterone, DHT, AR and metribolone regulated genes were strongly enriched and the respective pathways activated, whereas Bicalutamide and LY294002 regulated genes were downregulated (Figure 4C). Interestingly, among the overlapping, deregulated genes, 70.1% were counter‐regulated, suggesting antagonistic functions for CRYM and AR signaling. In this data set, 843 out of 3427 significantly deregulated genes overlapped with the CRYM deregulated genes (Figure 4D).

Next, we treated the LNCaP cells with T3, DHT or a combination and performed RNA‐Seq analysis. LNCaP cells kept in hormone free medium (HFM) (Charcoal‐stripped medium) were supplemented with T3, DHT or a combination thereof (HFM only, 10 nM T3, 10 nM DHT, 10 nM T3 + 10 nM DHT). T3 significantly induced *AR* expression and T3 supplementation led to an increase in expression of AR target gene *KLK3* (Figure S2A). The AR chaperon *CHKA* was induced in presence of DHT (Figure S2A). To further validate, LNCaP cells were incubated with increasing amounts of T3 (0, 1, 10, 100 nM) for 48 hours in HFM resulting PSA increase on protein and mRNA level (Figure S2B). Complementary to this approach, shRNA‐mediated knock‐down of CRYM in LNCaP cells resulted in a significant increase in PSA levels (38 ± 2 ng/mL as compared to 10 ± 2 ng/mL of control, Figure 4E). Since AR protein or mRNA levels were not altered in CRYM over‐expressing cells (Figure S2C), the strong effect of CRYM overexpression on target gene expression suggests a crosstalk between downstream targets of androgen and thyroid hormone signaling in PCa. For example, overexpression of CRYM led to a reduction of choline kinase α (CHKA) protein levels (Figure S2D)

### 3.5 CRYM alters the choline metabolism and metabolic shift caused by T3

**FIGURE 5:**
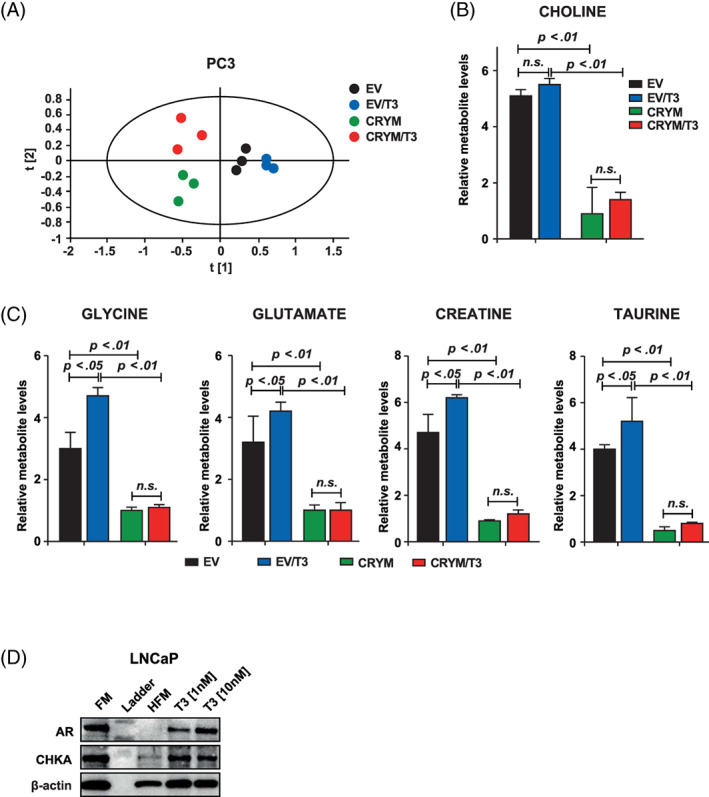
CRYM overexpression caused to the metabolic alterations in PCa. A, Score plot of partial least squares‐discriminant analysis (PLS‐DA) model of PC3 cell pellet extract fitted using 1H‐NMR spectral data. PC3 cells without CRYM overexpressing vector (right) were separated from PC3 cells with CRYM overexpressing vector (left) along the first component. B, Measurement of free choline in CRYM overexpression (*P* < .01) or EV in the presence of T3. C, Relative metabolite levels of glycine, glutamate, creatinine and taurine with and without T3 in LNCaP with overexpression of CRYM or EV measured by ^1^H‐NMR. D, Immunoblot analysis of AR and choline kinase α (CHKA) and β‐Actin as a control in RPMI 1640 medium with 10% FCS (FM), HFM and HFM with T3 supplementation (1 nM and 10 nM). CRYM, μ‐Crystallin; EV, empty vector; FCS, fetal calf serum; FM, full medium; PCa, prostate cancer [Color figure can be viewed at wileyonlinelibrary.com]

Recent studies have revealed that tumor suppressor pathways such as PTEN, p53 and RB are involved in regulation of glucose and glycine metabolism resulting in metabolic features that are unique for proliferative cancer cells. We took advantage of the 1H‐NMR‐based metabolomics to identify the role of CRYM in thyroid hormone signaling and metabolism during PCa progression. PC3 harboring empty vector or CRYM overexpression were stimulated with T3 for 48 hours. We performed intracellular and extracellular metabolite concentration analysis followed by PLS‐DA analysis, and found that T3 treatment or CRYM overexpression significantly affected the metabolic profile of PCa cells (Figure 5A). CRYM overexpression had a strong overall effect on the metabolome (represented by a shift along the t (1) axis in PLS‐DA) regardless of T3 stimulation. Our NMR metabolomic analysis showed that intracellular choline was significantly reduced upon CRYM overexpression in PC3 cells, whereas T3 led to a slight but nonsignificant choline increase (Figure 5B). Consequently, we found that CRYM(+) resulted in reduced expression of CHKA in PC3 cells (Figure S2D). Glycine, glutamate, creatine and taurine, which are known to be rapidly taken up by growing cancer cells, were increased by T3 treatment but reduced with CRYM overexpression (Figure 5C). CRYM overexpression did not substantially change the levels of metabolites in nontransformed RWPE‐1 epithelial prostate cells (Figure S3A). Multivariate analysis revealed that intracellular choline was not altered upon CRYM overexpression in RWPE‐1 cells, whereas T3 led to a slight increase in choline levels (Figure S3B). Glycine, glutamate, creatine and taurine also remained unaffected by CRYM overexpression (Figure S3C). Choline is an important component for phospholipid metabolism,^24^ and elevated choline is a metabolic hallmark of tumor progression and a valuable diagnostic biomarker in PCa.^24^, ^25^ Next we tested the effect of T3 on CHKA expression. Phosphorylation of choline by this enzyme is the first step of phosphatidylcholine synthesis. We found that T3 supplementation increased CHKA expression (Figure 5D). Vice versa we described above that CRYM suppressed FASN which is another essential component for membrane phospholipid synthesis. Of note, a recent report showed that CRYM knockout mice have increased body weight and PPΑRγ expression under high fat diet, adding a new aspect to the connection between CRYM and lipid metabolism.^25^ Taken together, our data demonstrate an inhibition of cancer‐associated metabolism profiles upon CRYM overexpression and suggest that CRYM expression could abolish the stimulating metabolic influence of T3 on PCa cells.

### 3.6 FMC‐PET [18F]fluoromethylcholine imaging of PCa patients is a surrogate marker for thyroid hormone action in PCa

**FIGURE 6:**
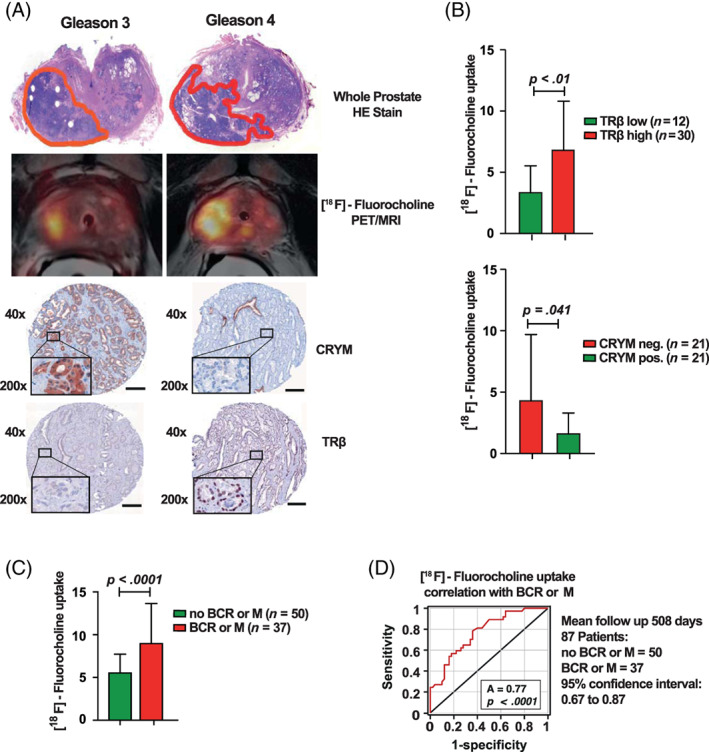
Noninvasive imaging using [^18^F]fluoromethylcholine (FMC) and PET is a surrogate marker for the activity of thyroid hormone metabolism in PCa. A, Hematoxylin Eosin (HE) whole mount sections of two different prostate cancer specimens on the left with a Gleason 3 lesion and on the right with a larger Gleason 4 lesion. Corresponding FMC PET/MRI of the same patient shows FMC uptake on the left (Gleason 3) and on the right (Gleason 4). IHC showing CRYM stainings positive in the Gleason 3 lesion on the left and TRβ stainings positive in the Gleason 4 lesion on the right (scale bar = 150 μm). B, Statistical evaluation of 42 PCa patients who underwent FMC PET/MRI prior to radical prostatectomy. CRYM and TRβ protein levels in tumors were analyzed using IHC. Choline uptake in tumor specimens with high TRβ expression (TRβ 2‐6 score; n = 30, *P* < .01) or no CRYM expression (n = 21, *P* = .041). C, Choline uptake correlated to BCR or metastases in receiver‐operating‐characteristics‐curve analysis (AUC = 0.77, *P* < .0001). D, 87 Patients with FMC PET/MRI and a mean follow‐up time of 508 days were divided into a group that developed BCR and/or metastasis (n = 37) and a group that did not (n = 50). The first group had significantly higher choline levels in FMC PET/MRI (*P* < .0001). AUC, area under the curve; BCR, biochemical recurrence; FMC, [18F]fluoromethylcholine; HE, hematoxylin eosin; IHC, immunohistochemistry; MRI, magnetic resonance imaging; PCa, prostate cancer; PET, positron emission tomography [Color figure can be viewed at wileyonlinelibrary.com]

Radiolabeled choline such as [18F]fluoromethylcholine (FMC) is widely used as a PET/MRI tracer for the evaluation of PCa progression. This is potentially due to the increased rate of lipid metabolism in neoplastic prostate tissues where choline is needed to generate phosphatidylcholine, an important component of the cell membrane.^26^ Having in mind the effect of CRYM on intracellular choline metabolism in PC3 PCa cells, CRYM and TRβ protein expression were analyzed by IHC in the same patient prostatectomy specimens. Figure 6A shows whole mount sections of two different PCa specimens along with the respective FMC‐PET results. A representative tumor with a Gleason pattern 3 (GL3) lesion displayed a lower FMC signal along with high CRYM/low TRβ expression (left panel) compared to a Gleason pattern 4 (GL4) PCa lesion with higher FMC signal and low CRYM/high TRβ expression (right panel). Next we measured FMC levels in a cohort of 42 PCa patients before they underwent radical prostatectomy. Statistical evaluation of 42 patients confirmed a direct correlation for PET‐FMC uptake signal to IHC TRβ levels and an inverse correlation to IHC CRYM levels in the respective tumor tissue (Figure 6B). A follow‐up analysis was performed in 87 patients that correlated with FMC uptake to BCR and/or synchronous metastatic disease. Mean follow up time in this cohort was 508 days. Patients with BCR or already initial metastases had significantly higher FMC uptake (Figure 6C). Receiver operating characteristic analysis shows the confidence interval curve of 0.77 with *P* < .0001 (Figure 6D). These data indicate that FMC signal in prostate is indicative of BCR, and that this correlates with TRβ and CRYM expression. Our results suggest that choline is closely associated with intracellular thyroid hormone levels and that the ^18^F‐FMC PET/MRI tracer for the activity of thyroid hormone could be used to predict high and low risks in PCa patients. Whether FMC‐PET imaging can be used as a marker for the activity of thyroid hormone metabolism in PCa needs to be tested in further studies. In summary, we conclude that the absence of CRYM results in an increased choline metabolism in PCa, which is a poor prognostic indicator that can noninvasively be measured in vivo by^18^F‐FMC PET/MRI.

## 4 DISCUSSION

We investigated the crosstalk of CRYM with T3 and AR signaling in PCa. We demonstrated that the growth‐promoting effect of T3 could be attenuated by the intracellular thyroid hormone binding protein CRYM. Increased CRYM expression resulted in decreased availability of T3, significantly prolonged time to BCR and suppressed important targets of the androgen‐regulated gene network in PCa. Loss of CRYM in aggressive PCa cells led to a massive increase in PSA. We show here for the first time a tumor suppressive effect of CRYM by reducing choline metabolism in PCa. In FMC‐PET/MRT studies of PCa patients we found a significant correlation of high CRYM expression with low choline uptake. Furthermore, we show a significant inverse correlation between CRYM, AR and TRβ in human PCa patient cohorts. Expression of CRYM may be a novel biomarker for the diagnosis and prognosis of PCa. We propose that targeting the thyroid hormone pathway is a rational strategy to treat aggressive PCa.

Besides androgens, some other factors such as thyroid or estrogen hormones have been proposed to take part in the progression of PCa.^27^, ^28^ Recent epidemiological studies have linked high thyroid hormone levels to higher incidence of PCa suggesting a tumor promoting role of T3 in PCa.^29^ However, underlying molecular mechanisms in which thyroid hormone signaling contributes to tumor growth are still not well understood.

The growth‐promoting effect of T3 can be attenuated by the intracellular thyroid hormone binding protein CRYM. In Graves' hyperthyroidism, which is an autoimmune disease five times more abundant in females than in males, anti‐thyroid medication with methimazole results in CRYM mRNA overexpression which suggests an inverse correlation of CRYM with thyroid hormone.^30^ Increased levels of CRYM lead to suppression of important targets of the androgen‐regulated gene network including PSA. We found high CRYM levels in normal human prostate samples, decreased CRYM in PCa samples and CRYM expression could not be detected in metastatic PCa. Patients with high CRYM levels in their PCa cells had a significantly prolonged time to BCR, suggesting CRYM as a positive predictive marker in PCa. Since CRYM is involved in sequestration of T3, the loss of thyroid hormone binding protein CRYM in PCa could result in increased thyroid hormone activity. Our data on CRYM/T3 regulation further support the idea that CRYM acts not only as a binding protein but also as a T3 sink in the cytoplasm. CRYM overexpression downregulated thyroid and androgen regulated genes, which indicates a possible interaction of thyroid and androgen signaling pathways in PCa. High CRYM levels could possibly antagonize the impact of thyroid hormone on androgen signaling.

PET imaging using metabolic tracers offers the opportunity not only for noninvasive diagnostics but also long‐term surveillance. Interestingly, we found that overexpression of T3‐buffering CRYM leads to a reduction in intracellular choline levels in PC3 cells. CHKA has a crucial role in phospholipid metabolism through catalyzing the choline phosphorylation to form phosphocholine, a phospholipid component of bilayers in cell membranes.^31^ Total choline expression has been shown to be elevated in PCa tissue as compared to healthy, age matched controls.^32^, ^33^, ^34^ This feature has been successfully used in combination with PET/MRI to enhance the accuracy of noninvasive diagnostics.^35^ The clinical outcome of PCa is highly variable reflecting the genetic and pathophysiologic heterogeneity of the disease. Therefore, biomarkers for detection of aggressive tumors are highly warranted. The results of our study contribute to define aggressive PCa cases and suggest a novel targeting approach for CRPC. Of note, expression of CRYM could be applied not only for prognostic risk but also as a biomarker to guide patient care and management. Our FMC‐PET/MRT cohort encompassed 42 patients displaying a significant correlation between CRYM and TRβ status. It is clear that larger, independent patient series are needed before clinical applicability of our findings in the context of FMC‐PET/MRT would be possible. In addition, PCa screening based on serum PSA measurements shows limited sensitivity and specificity. Thus, it remains to be determined if FMC‐PET/MRT could be used to noninvasively discriminate early stage PCa from nonmalignant conditions associated with serum PSA increase. In addition, our data now provide a mechanistic rationale for this diagnostic technique, suggesting that choline metabolism is tightly linked to intracellular thyroid hormone status. For treatment of malignancies, propylthiouracil (PTU), a thyroid hormone synthesis blocker that generates a hypothyroid state, was shown to reduce tumor growth of engrafted lung and PCa cells in NCR‐Mice.^7^ Hypothyroidism was shown to slow down growth of aggressive breast cancer.^36^ Earlier reports also indicated that hypothyroidism induced by PTU in rats inhibited metastatic growth, formed smaller tumors and prolonged survival in liver cancer.^37^ The question of how hypothyroidism diminishes growth of PCa could be possibly addressed in the future using more specific drugs with less side effects to block the thyroid hormone pathway, potentially in combination with AR blockers.^38^, ^39^

In conclusion, we provide strong evidence for the protective effect of T3 buffering by CRYM in PCa. Therefore, CRYM might represent a novel biomarker for detection of good prognostic PCa. In addition, we provide a rationale for how thyroid hormone metabolism could influence choline levels in PCa, which could be highly relevant for diagnostic imaging techniques. Based on the fact that thyroid hormone signaling might act as a new oncogenic factor, our study also contributes to the understanding of aggressive PCa. Importantly, new treatment avenues using novel and specific antithyroid drugs may open up, and inclusion of such drugs in currently used regimens for metastatic PCa will be of interest.

## CONFLICT OF INTEREST

The authors declare no potential conflict of interest.

## ETHICS STATEMENT

All human samples for TMA and their use in this study were approved by the Research Ethics Committee of the Medical University Vienna, Austria (1248/2015) and by the Research Ethics Committee for Germany (395/2008BO1) (Bonn, Germany). For the FMC‐PET/MRI patient study, a prospective clinical phase III trial (NCT02659527, EudraCT No.: 2014‐004758‐33), which was approved by the local Ethics committee (1985/2014) and the national drug authorities.

## Supporting information


**Figure S1** **(A)** *CRYM* mRNA expression levels in PCa patients, analyzed from primary tumor (PCa), metastases or normal prostate gland samples (left) and primary tumor (PCa) samples or metastases (right). Data were extracted from the Oncomine Platform using the following studies: Varambally Prostate (left: normal (n = 6), PCa (n = 7); *P = 0.002* and metastases (n = 6); *P = 0.00073*) and LaTulippe Prostate (right: PCa (n = 23), metastases (n = 9); *P = 0.004*). **(B)** *CRYM* mRNA expression levels in PCa patients, analyzed from primary prostate adenocarcinoma samples with Gleason scores 6 and 9 (left), primary prostate carcinoma samples with Gleason scores 6 and 7 (middle) or primary prostate carcinoma samples with stages N0 and N1+ (right). All data were obtained from the Oncomine Platform from the following studies: Vanaja Prostate (left: Gleason score 6 (n = 12), Gleason score 9 (n = 15); *P = 0.00024*), Luo Prostate 2 (middle: Gleason score 6 (n = 3), Gleason score 7 (n = 7); *P = 0.007*) and LaTulippe Prostate (right: Pathological grade N0 (n = 19), N1+ (n = 4); *P = 0.015*). **(C)** Immunoblot analysis of AR, KLK3 and TRβ in PCa cell lines RWPE‐1, LNCaP, LAPC4, VCAP, 22Rv1, PC3 and DU145. β‐Actin was used as loading control. **(D)** T3 uptake measured in the growth medium of LNCaP cells transfected with empty vector or CRYM(+) using an electrochemiluminescence immune assay at day 1 and 2. **(E)** PC3 cells were transfected with empty vector or CRYM(+) and the number of cells were quantified at day 2 and 4 (*P = 0.0004*).
**Figure S2. (A)** LNCaP cells used for RNA‐Seq analysis were incubated in HFM (Charcoal stripped medium) supplemented with T3, DHT or in combination (HFM only, 10 nM T3, 10 nM DHT, 10 nM T3 + 10 nM DHT). Results for androgen target genes AR, KLK3 and CHKA are shown. **(B)** Western blot shows PSA expression in LNCaP cells that were incubated with increasing amounts of T3 (0, 1, 10, 100 nM) for 48 hours. Cells were starved in serum‐free medium for 24 hours prior to thyroid hormone incubation. qPCR shows PSA (*KLK3*) mRNA expression in LNCaP cells that were treated with T3 (50 nM), DHT (10 nM) and in combination for 48 hours in Charcoal stripped medium. **(C)** AR expression in EV or CRYM‐overexpressing LNCaP cells at the protein (immunoblot) and the mRNA (RNA‐seq) level. **(D)** Immunoblot of CRYM and CHKA expression in PC3 cells that were transiently transfected with EV or CRYM(+).
**Figure S3. (A)** Score plot of partial least squares‐discriminant analysis (PLS‐DA) model of RWPE‐1 cell pellet extract fitted using NMR spectral data. The cell pellets were extracted using methanol. RWPE‐1 cells with T3 treatment were separated from those without T3 treatment along the first component. The RWPE‐1 cells with CRYM vector were separated from those without CRYM vector along the second component. **(B)** Measurement of free choline in CRYM overexpression (n.s.) or EV in the absence or presence of T3. **(C)** Relative metabolite levels of glycine, glutamate, creatinine and taurine with and without T3 in RWPE‐1 with overexpression of CRYM or EV measured by ^1^H‐NMR.
**Figure S4.** Densitometric analysis of proteins normalized to the β‐actin using Image J software. **(A)** CRYM relative density in PCa cell panel (WB: Figure 2A). **(B)** CRYM overexpression relative density in PC3 and DU145 cells and TRβ level in the same setting (WB: Figure 2B). **(C)** CRYM monomeric and dimeric relative density in CRYM overexpressed LNCaP cells (WB: Figure 4A). CRYM relative density in CRYM knockeddown LNCaP cells (WB: Figure 4D). **(D)** AR relative density in T3‐treated LNCaP cells (WB: Figure 5D). **(E)** AR, TRβ and PSA (KLK3) relative density in PCa cell panels (WB: Figure S1C). **(F)** PSA (KLK3) relative density in T3‐treated LNCaP cells (WB: Figure S2B). **(G)** AR relative density in CRYM overexpressed LNCaP cells (WB: Figure S2C). **(H)** CRYM and CHKA relative density in CRYM overexpressed PC3 cells (WB: Figure S2D).

**Supplementary Table** List of deregulated genes by CRYM overexpression

## Data Availability

The data that support the findings of this study are available from the corresponding authors upon reasonable request. The CRYM RNA‐seq data (GSE101525) that support the findings of study are openly available from the NCBI database at: (https://www.ncbi.nlm.nih.gov/geo/query/acc.cgi?acc=GSE101525).
