# Supplementary material for: Thyroid and androgen receptor signaling are antagonized by μ‐Crystallin in prostate cancer
Source: Int J Cancer. 2020 Oct 31;148(3):731–47. doi: 10.1002/ijc.33332 (PMC7756625; doi:10.1002/ijc.33332)
Supplement: Supplementary file 1 — Figure S1 (A) CRYM mRNA expression levels in PCa patients, analyzed from primary tumor (PCa), metastases or normal prostate gland samples (left) and primary tumor (PCa) samples or metastases (right). Data were extracted from the Oncomine Platform using the following studies: Varambally Prostate (left: normal (n = 6), PCa (n = 7); P = 0.002 and metastases (n = 6); P = 0.00073) and LaTulippe Prostate (right: PCa (n = 23), metastases (n = 9); P = 0.004). (B) CRYM mRNA expression levels in PCa patients, analyzed from primary prostate adenocarcinoma samples with Gleason scores 6 and 9 (left), primary prostate carcinoma samples with Gleason scores 6 and 7 (middle) or primary prostate carcinoma samples with stages N0 and N1+ (right). All data were obtained from the Oncomine Platform from the following studies: Vanaja Prostate (left: Gleason score 6 (n = 12), Gleason score 9 (n = 15); P = 0.00024), Luo Prostate 2 (middle: Gleason score 6 (n = 3), Gleason score 7 (n = 7); P = 0.007) and LaTulippe Prostate (right: Pathological grade N0 (n = 19), N1+ (n = 4); P = 0.015). (C) Immunoblot analysis of AR, KLK3 and TRβ in PCa cell lines RWPE‐1, LNCaP, LAPC4, VCAP, 22Rv1, PC3 and DU145. β‐Actin was used as loading control. (D) T3 uptake measured in the growth medium of LNCaP cells transfected with empty vector or CRYM(+) using an electrochemiluminescence immune assay at day 1 and 2. (E) PC3 cells were transfected with empty vector or CRYM(+) and the number of cells were quantified at day 2 and 4 (P = 0.0004). Figure S2. (A) LNCaP cells used for RNA‐Seq analysis were incubated in HFM (Charcoal stripped medium) supplemented with T3, DHT or in combination (HFM only, 10 nM T3, 10 nM DHT, 10 nM T3 + 10 nM DHT). Results for androgen target genes AR, KLK3 and CHKA are shown. (B) Western blot shows PSA expression in LNCaP cells that were incubated with increasing amounts of T3 (0, 1, 10, 100 nM) for 48 hours. Cells were starved in serum‐free medium for 24 hours prior to thyroid hormone incu [file IJC-148-731-s001.pdf]

SUPPLEMENTARY MATERIAL

Thyroid and androgen receptor signaling are antagonized by CRYM in prostate cancer

Osman Aksoy, Jan Pencik, Markus Hartenbach, Ali A. Moazzami, Michaela Schlederer, Theresa Balber, Adam Varady, Cecile Philippe, Pascal A. Baltzer, Bismoy Mazumder, Jonathan B Whitchurch, Christopher J. Roberts, Andrea Haitel, Merima Herac, Martin Susani, Markus Mitterhauser, Rodrig Marculescu, Judith Stangl-Kremser, Melanie R. Hassler, Gero Kramer, Shahrokh F. Shariat, Suzanne D. Turner, Boris Tichy, Jan Oppelt, Sarka Pospisilova, Sabrina Hartenbach, Simone Tangermann, Gerda Egger, Heidi A. Neubauer, Richard Moriggl, Zoran Culig, Georg Greiner, Gregor Hoermann, Marcus Hacker, David M. Heery, Olaf Merkel and Lukas Kenner

Table of contents

Supplementary figures:

Supplementary figure 1 (Figure S1)

Supplementary figure 2 (Figure S2)

Supplementary figure 3 (Figure S3)

Supplementary figure 4 (Figure S4)

Supplementary table:

List of deregulated genes by CRYM overexpression

Figure S1

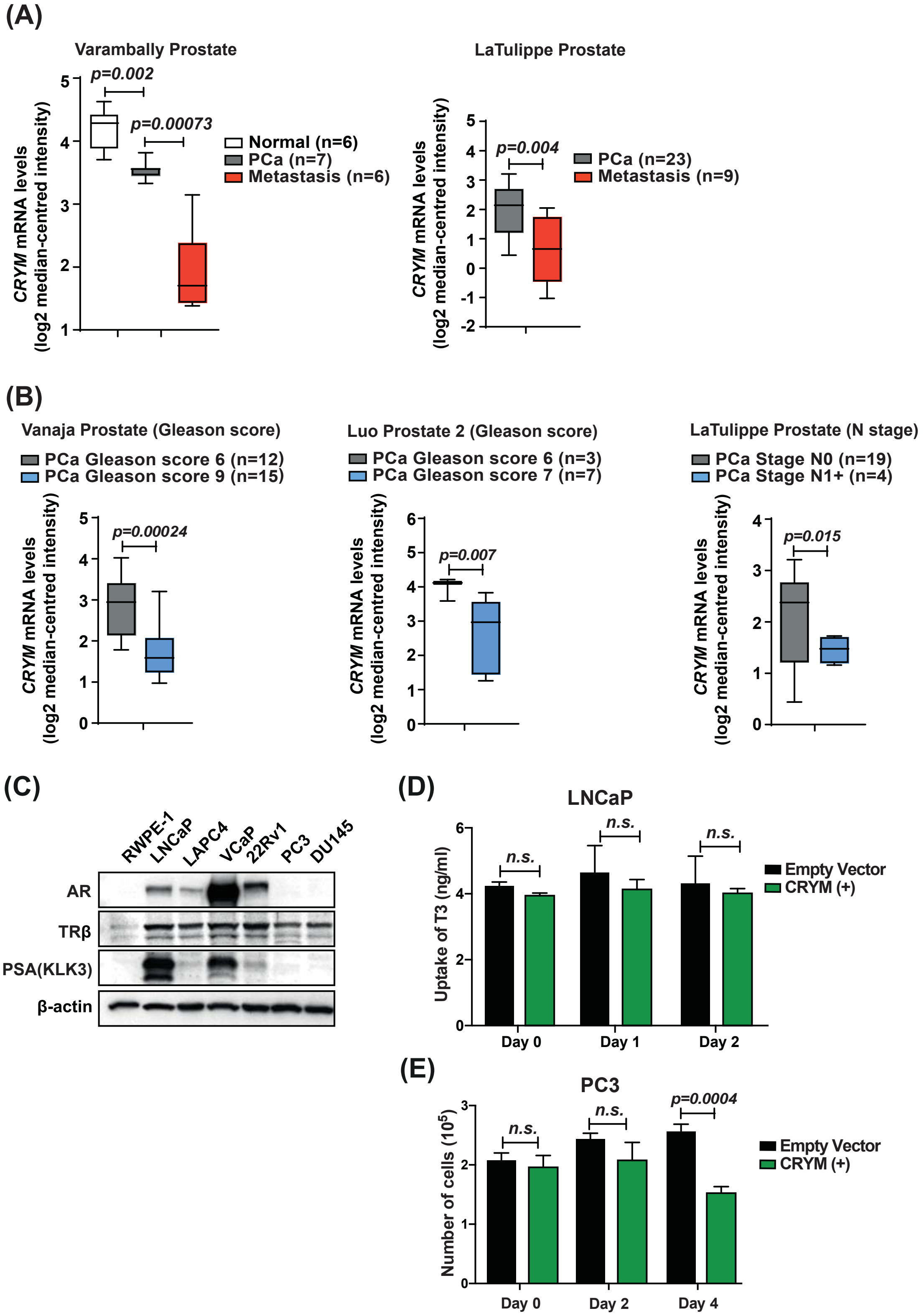
(A)
Varambally Prostate
p=0.002
p=0.00073
Normal (n=6)
PCa (n=7)
Metastasis (n=6)
CRYM mRNA levels (log2 median-centred intensity)
LaTulippe Prostate
p=0.004
PCa (n=23)
Metastasis (n=9)
(B)
Vanaja Prostate (Gleason score)
PCa Gleason score 6 (n=12)
PCa Gleason score 9 (n=15)
p=0.00024
Luo Prostate 2 (Gleason score)
PCa Gleason score 6 (n=3)
PCa Gleason score 7 (n=7)
p=0.007
LaTulippe Prostate (N stage)
PCa Stage N0 (n=19)
PCa Stage N1+ (n=4)
p=0.015
(C)
RWPE-1
LNCaP
LAPC4
VCaP
22Rv1
PC3
DU145
AR
TRβ
PSA(KLK3)
β-actin
(D)
LNCaP
Uptake of T3 (ng/ml)
n.s.
n.s.
n.s.
Empty Vector
CRYM (+)
Day 0
Day 1
Day 2
(E)
PC3
Number of cells (10^5)
n.s.
n.s.
p=0.0004
Empty Vector
CRYM (+)
Day 0
Day 2
Day 4

Figure S1. (A) *CRYM* mRNA expression levels in PCa patients, analyzed from primary tumor (PCa), metastases or normal prostate gland samples (left) and primary tumor (PCa) samples or metastases (right). Data were extracted from the Oncomine™ Platform using the following studies: Varambally Prostate (left: normal (n=6), PCa (n=7); *p=0.002* and metastases (n=6); *p=0.00073*) and LaTulippe Prostate (right: PCa (n=23), metastases (n=9); *p=0.004*). **(B)** *CRYM* mRNA expression levels in PCa patients, analyzed from primary prostate adenocarcinoma samples with Gleason scores 6 and 9 (left), primary prostate carcinoma samples with Gleason scores 6 and 7 (middle) or primary prostate carcinoma samples with stages N0 and N1+ (right). All data were obtained from the Oncomine™ Platform from the following studies: Vanaja Prostate (left: Gleason score 6 (n=12), Gleason score 9 (n=15); *p=0.00024*), Luo Prostate 2 (middle: Gleason score 6 (n=3), Gleason score 7 (n=7); *p=0.007*) and LaTulippe Prostate (right: Pathological grade N0 (n=19), N1+ (n=4); *p=0.015*). **(C)** Immunoblot analysis of AR, KLK3 and TRβ in PCa cell lines RWPE-1, LNCaP, LAPC4, VCAP, 22Rv1, PC3 and DU145. β-Actin was used as loading control. **(D)** T3 uptake measured in the growth medium of LNCaP cells transfected with empty vector or CRYM(+) using an electrochemiluminescence immune assay at day 1 and 2. **(E)** PC3 cells were transfected with empty vector or CRYM(+) and the number of cells were quantified at day 2 and 4 (*p=0.0004*).

Figure S2

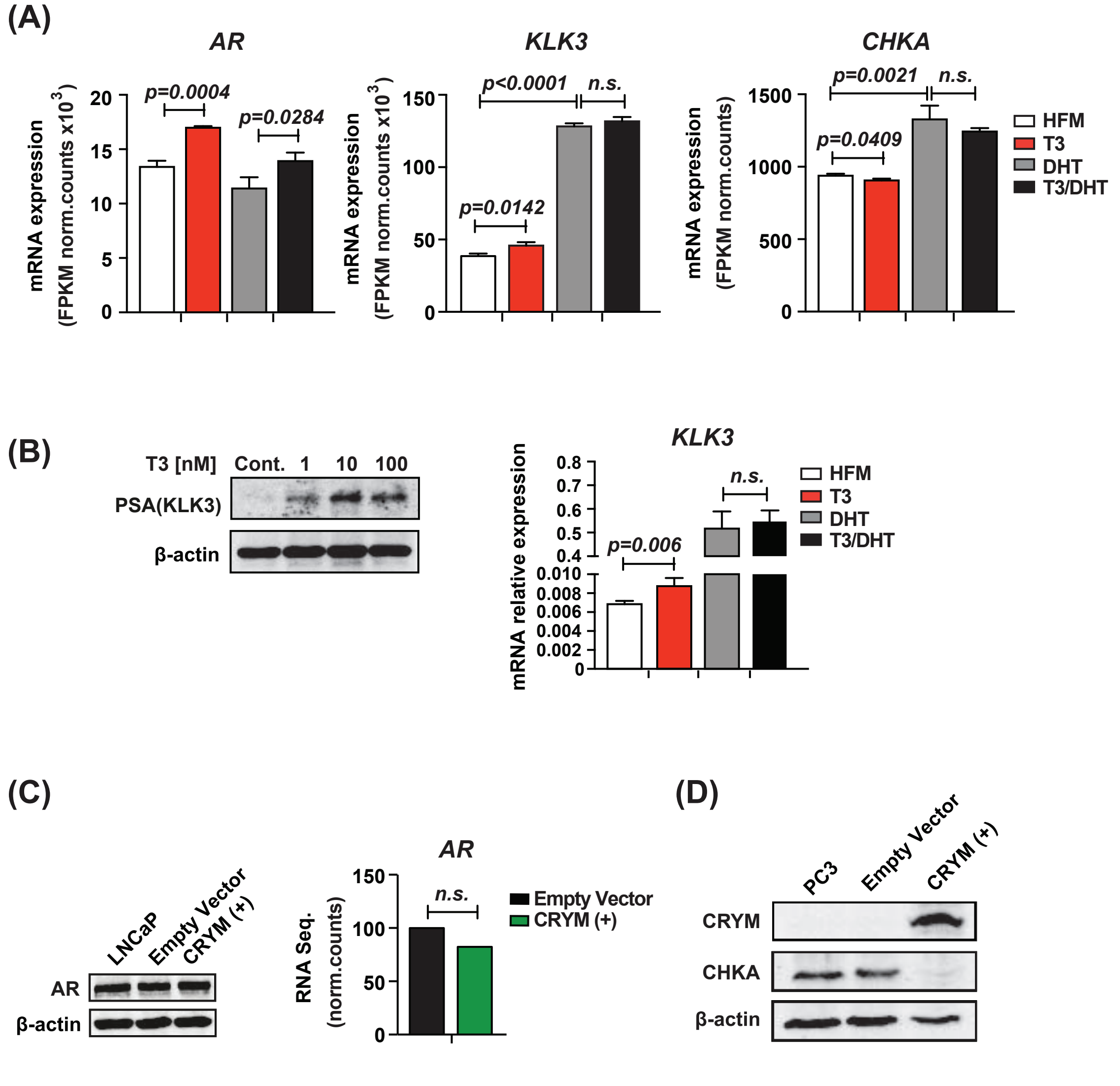

Figure S2. (A) LNCaP cells used for RNA-Seq analysis were incubated in HFM (Charcoal stripped medium) supplemented with T3, DHT or in combination (HFM only, 10 nM T3, 10 nM DHT, 10 nM T3 + 10 nM DHT). Results for androgen target genes AR, KLK3 and CHKA are shown. **(B)** Western blot shows PSA expression in LNCaP cells that were incubated with increasing amounts of T3 (0, 1, 10, 100 nM) for 48 hours. Cells were starved in serum-free medium for 24 hours prior to thyroid hormone incubation. qPCR shows PSA (*KLK3*) mRNA expression in LNCaP cells that were treated with T3 (50 nM), DHT (10nM) and in combination for 48 hours in Charcoal stripped medium. **(C)** AR expression in EV or CRYM-overexpressing LNCaP cells at the protein (immunoblot) and the mRNA (RNA-seq) level. **(D)** Immunoblot of CRYM and CHKA expression in PC3 cells that were transiently transfected with EV or CRYM(+).

Figure S3

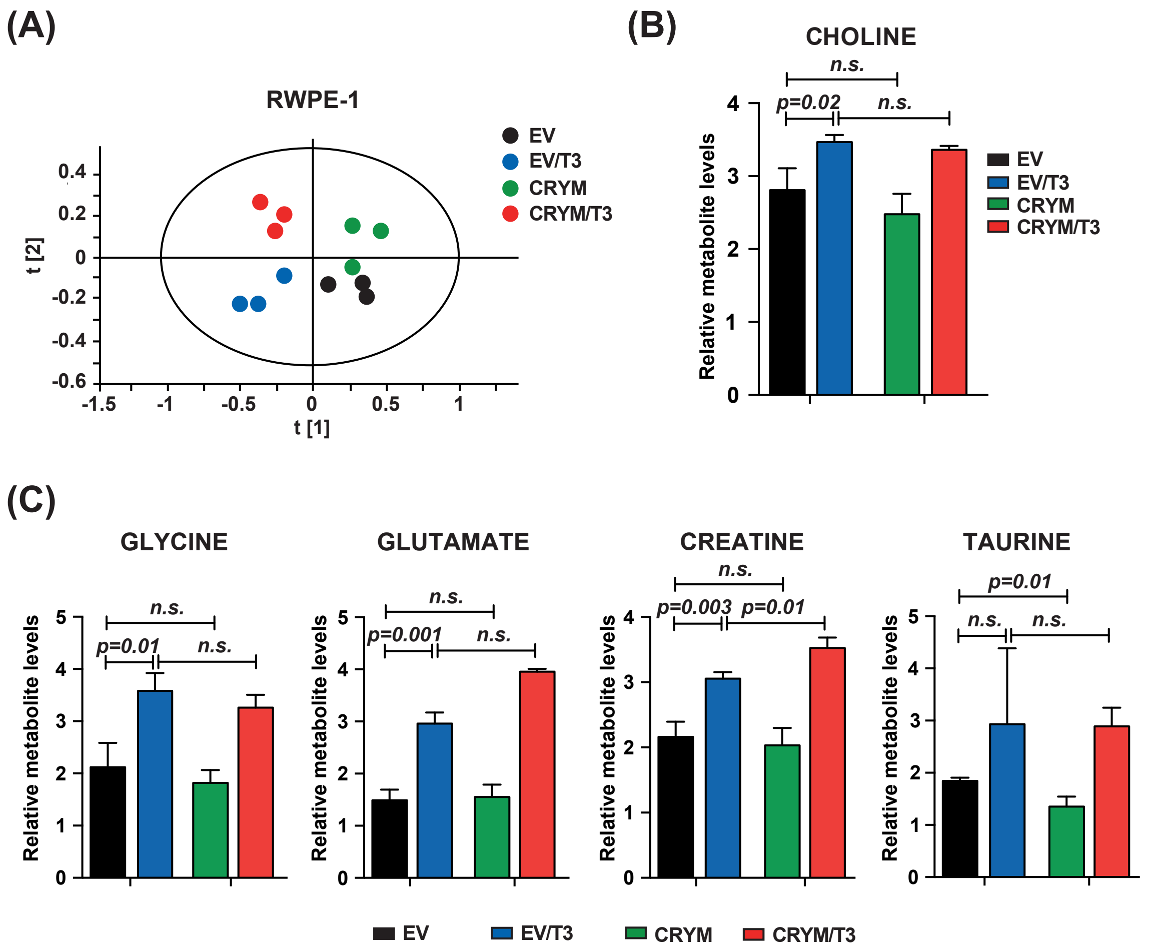

Figure S3. (A) Score plot of partial least squares-discriminant analysis (PLS-DA) model of RWPE-1 cell pellet extract fitted using NMR spectral data. The cell pellets were extracted using methanol. RWPE-1 cells with T3 treatment were separated from those without T3 treatment along the first component. The RWPE-1 cells with CRYM vector were separated from those without CRYM vector along the second component. **(B)** Measurement of free choline in CRYM overexpression (n.s.) or EV in the absence or presence of T3. **(C)** Relative metabolite levels of glycine, glutamate, creatinine and taurine with and without T3 in RWPE-1 with overexpression of CRYM or EV measured by ^{1}H-NMR.

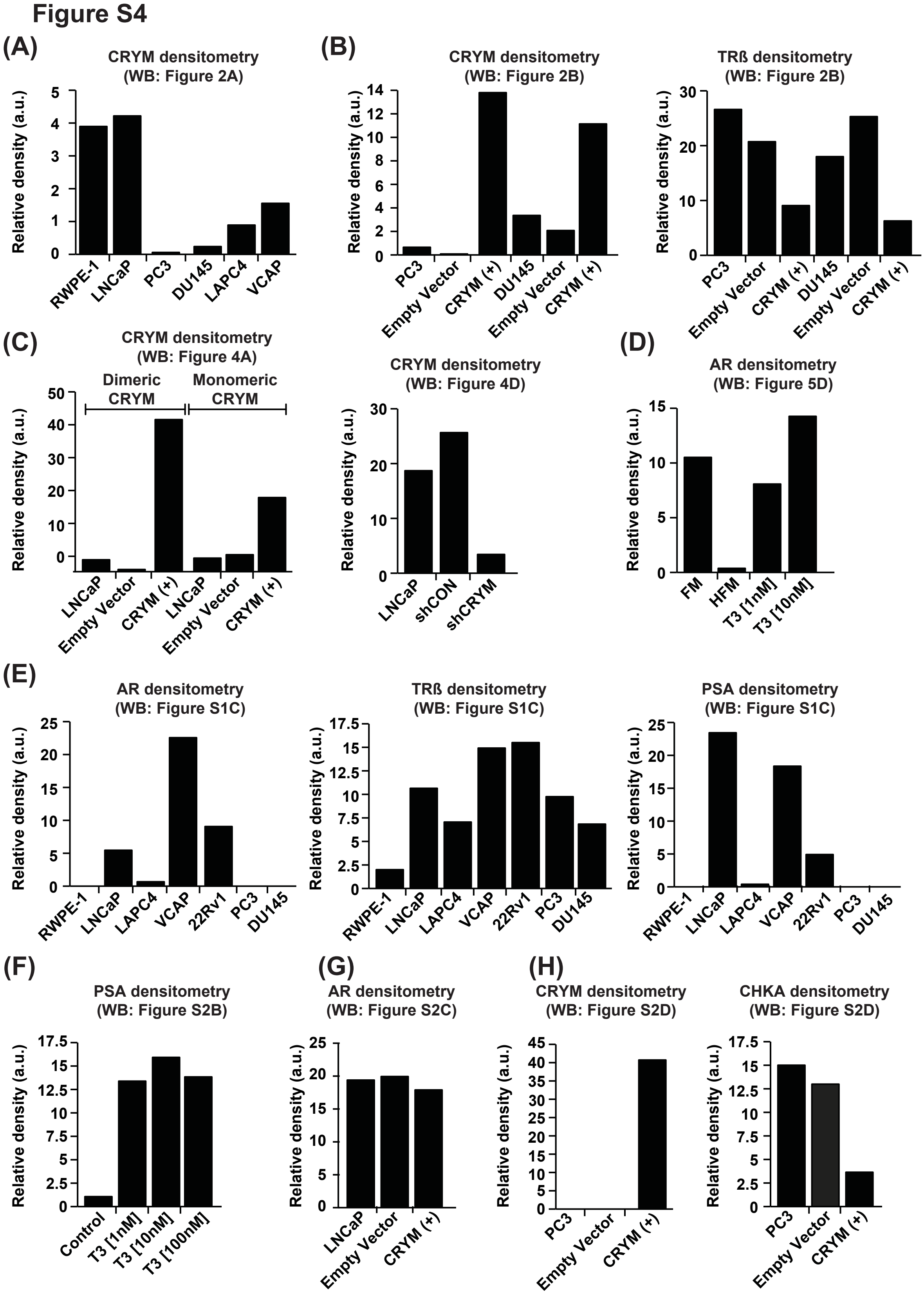
Figure S4
(A)
CRYM densitometry
(WB: Figure 2A)
Relative density (a.u.)
RWPE-1
LNCaP
PC3
DU145
LAPC4
VCAP
(B)
CRYM densitometry
(WB: Figure 2B)
PC3
Empty Vector
CRYM (+)
DU145
Empty Vector
CRYM (+)
TRß densitometry
(WB: Figure 2B)
(C)
CRYM densitometry
(WB: Figure 4A)
Dimeric CRYM
Monomeric CRYM
CRYM densitometry
(WB: Figure 4D)
LNCaP
shCON
shCRYM
(D)
AR densitometry
(WB: Figure 5D)
FM
HFM
T3 [1nM]
T3 [10nM]
(E)
AR densitometry
(WB: Figure S1C)
TRß densitometry
(WB: Figure S1C)
PSA densitometry
(WB: Figure S1C)
22Rv1
(F)
PSA densitometry
(WB: Figure S2B)
Control
T3 [100nM]
(G)
AR densitometry
(WB: Figure S2C)
(H)
CRYM densitometry
(WB: Figure S2D)
CHKA densitometry
(WB: Figure S2D)

Figure S4. Densitometric analysis of proteins normalized to the β-actin using Image J software. **(A)** CRYM relative density in PCa cell panel (WB: fig. 2A). **(B)** CRYM overexpression relative density in PC3 and DU145 cells and TRβ level in the same setting (WB: fig. 2B). **(C)** CRYM monomeric and dimeric relative density in CRYM overexpressed LNCaP cells (WB: fig. 4A). CRYM relative density in CRYM knocked-down LNCaP cells (WB: fig. 4D). **(D)** AR relative density in T3-treated LNCaP cells (WB: fig. 5D). **(E)** AR, TRβ and PSA (KLK3) relative density in PCa cell panels (WB: fig. S1C). **(F)** PSA (KLK3) relative density in T3-treated LNCaP cells (WB: fig. S2B). **(G)** AR relative density in CRYM overexpressed LNCaP cells (WB: fig. S2C). **(H)** CRYM and CHKA relative density in CRYM overexpressed PC3 cells (WB: fig. S2D).
